# Effects of Heat Stress on Goat Production and Mitigating Strategies: A Review

**DOI:** 10.3390/ani14121793

**Published:** 2024-06-14

**Authors:** Felix Danso, Lukman Iddrisu, Shera Elizabeth Lungu, Guangxian Zhou, Xianghong Ju

**Affiliations:** 1 College of Coastal Agricultural Sciences, Guangdong Ocean University, Zhanjiang 524088, China; fdanfreeman@gmail.com (F.D.); lungushera@gmail.com (S.E.L.); 2 Department of Veterinary Medicine, Guangdong Ocean University, Zhanjiang 524088, China; 3 Guangdong Provincial Key Laboratory of Aquatic Product Processing and Safety, Guangdong Provincial Engineering Technology, Research Center of Marine Food, Key Laboratory of Advanced Processing of Aquatic Products of Guangdong Higher Education Institution, College of Food Science and Technology, Guangdong Ocean University, Zhanjiang 524088, China; lukmaniddrisu54@gmail.com

**Keywords:** heat stress, goat production, insulin-like growth factor-1, behavioral characteristics, palpitating

## Abstract

**Simple Summary:**

This review highlights the various adverse effects of heat stress on the production and general well-being of the goat industry. Goats possess exceptional adaptability traits, allowing them to thrive well in diverse environments, particularly in harsh conditions such as tropical and semi-arid zones. This makes goats the ideal small ruminants for food security in an era of inevitable and continuous climate change. Despite the inherent ability of goats to withstand extreme weather conditions, their reproductivity and overall productivity are compromised in the event of heat stress. Therefore, gaining in-depth knowledge of the effects of heat stress and the various mitigating strategies to alleviate heat stress is a significant approach to ensuring food security. These efforts will serve as a benchmark for genetically engineering heat-tolerant goats suitable for the current climatic conditions to ensure optimal productivity in the future.

**Abstract:**

Goats, versatile creatures selectively bred for various purposes, have become pivotal in shaping the socioeconomic landscape, particularly in rural and economically challenged areas. Their remarkable ability to withstand and adapt to extreme heat has proven invaluable, allowing them to flourish and reproduce in even the harshest climates on Earth. Goat farming has emerged as a reliable and sustainable solution for securing food resources. However, despite its significance, the goat-producing industry has received less attention than other ruminants. Despite goats’ inherent resilience to heat, their productivity and reproductive performance suffer under high ambient temperatures, leading to heat stress. This presents a significant challenge for goat production, necessitating a comprehensive multidisciplinary approach to mitigating the adverse effects of heat stress. This review aims to explore the diverse impacts of heat stress on goats and propose effective measures to address the sector’s challenges. By understanding and addressing these issues, we can enhance the resilience and sustainability of goat farming, ensuring its continued contribution to food security and socioeconomic development.

## 1. Introduction

Small ruminants have a significant economic impact worldwide as they are crucial for the livelihoods of millions of people who rear these animals in various climates [1,2,3]. Being widely distributed, goats have played a vital role in the agricultural revolution and the development of human existence. Goats (*Capra hircus*) are believed to have been accustomed to living, approximately thousands of years ago [4,5], in the region known as the ‘Fertile Crescent’ in the Middle East [6]. These animals significantly impact the ability of underprivileged families, particularly those in rural areas, to support themselves [7,8]. Goats are highly valued for their versatility in providing many resources, such as meat, milk, offal, hides, horn, muck for fuel, and fiber [3,4]. Furthermore, these animals exhibit exceptional adaptability to many geographical and climatic conditions, including extreme and challenging climates [9], surpassing the performances of other domesticated ruminants. Goats exhibit a higher level of heat tolerance than other species [10,11]. Globally, approximately 600 distinct goat breeds exist [4], each exhibiting varying abilities to adapt to different climatic conditions. According to Kojo [10], goats with slack skin and floppy ears have a higher tolerance to heat than other goats. Angora goats have a reduced capacity to cope with heat stress (HS) than other goat breeds. Despite their exceptional tolerance, the heat-dissipation efficiency of these animals frequently dwindles because of heat stress [9]. Hence, selecting an appropriate breed is essential for maintaining animal output in an increasingly challenging environment [12]. However, introducing a high-yielding species from temperate regions to desert and tropical locations has often failed, owing to inadequate adaptation to thermal stress. Interbreeding is an ideal approach to solving this menace.

Stress refers to a harmful impact exerted by several factors on the well-being and functioning of animals. It can also be ascribed as the intensity of extraneous influences that disrupt a body’s normal condition [11]. Animals experience a range of stressors, including physical, nutritional, chemical, psychological, and heat/thermal stress. Thermal stress is the most significant concern in rapidly changing climates [13]. It is a major stressor, particularly in tropical, subtropical [14], arid [12], and semi-arid [11,15] regions worldwide. Heat stress refers to the subjective discomfort and physiological load that occur when an individual is exposed to an extremely hot environment [1]. Livestock generates heat through the metabolic breakdown of feed for essential physiological functions such as lactation, gestation, growth, and maintenance. As the amount of feed consumed increases, the need to dissipate more heat increases. Heat stress is common in most livestock species, particularly dairy animals, as they require significant amounts of energy to maintain milk production. Both lactating dairy and meat goats fed for growth are prone to thermal stress. Awareness of physiological indicators, such as panting or increased water consumption, might enhance the visibility of heat stress. The economic consequences of heat stress in dairy cattle mostly manifest as decreased feed intake, reduced milk output, and minimum levels of solids (non-fat) [16]. Nevertheless, the reproductive process was also impaired. Ingraham et al. [17] discovered a correlation between heat stress and decreased conception rates. They found that as the average daily temperature humidity index (THI) rose from 70 to 84, the conception rate dropped significantly from 55% to 10%. Goats are more suitable for hot conditions than cattle because they have a smaller body mass, require less feed for upkeep, and have a higher digestive efficiency [18]. However, goats are still susceptible to heat stress, and factors such as breed-specific variances in the sweating rate and coat thickness can affect the level of heat stress experienced by goats. Small ruminant owners worldwide face the major and recurring challenge of climate change, which negatively impacts the productivity and health of these animals [13]. Environmental elements including high ambient temperature, sun radiation, and humidity directly and indirectly impact the animals’ welfare [19]. The ability of an animal to conserve energy, thermal, water, hormonal, and mineral balances is significantly hindered by high ambient temperatures [12]. The goal of this study is to evaluate the various consequences of heat stress on goats and to suggest effective approaches to alleviate the drawbacks of the industry.

## 2. Materials and Methods

### 2.1. Animal Model

Goats are globally distributed and have played a vital role in the agricultural revolution and development of human existence. Goats are believed to have been domesticated thousands of years ago in the Middle East and are now widely distributed because of their exceptional adaptability to many geographical locations, particularly extreme climatic zones. Globally, there are approximately 600 distinct goat breeds [4], each exhibiting varying abilities to adapt to different climatic conditions. Goat production has a significant economic impact as it plays an integral role in the livelihood of millions of people who rear them. Therefore, goats are an indispensable agricultural animal product. Heat stress is a limiting factor in animal production, and goats are no exception, despite their inherent ability to tolerate heat stress and thrive under diverse climatic conditions. The adverse effects of HS are discussed in this review using the existing published literature.

### 2.2. Heat Stress in Goat Production

Stressors are defined as the intensity of extraneous influences that disrupt a body’s normal condition. They are composed of physical, nutritional, chemical, psychological, and heat stresses, with heat stress being the most significant concern in these challenging climates. In this review, heat stress is defined as the subjective discomfort and physiological load that occurs when an individual is exposed to an extremely hot environment. Heat is generated in livestock through the metabolic breakdown of feed for essential physiological functions such as lactation, gestation, growth, and maintenance. Assembling in shaded areas, excessive salivation, rapid breathing with an open mouth, reduced feed intake, and increased water intake are visible behavioral characteristics ascribed to goats under heat stress. The effects of heat stress were categorized into nine (9) parameters of animal productivity and reproductive welfare. The nine (9) parameters considered in this review were behavioral response, growth and development, reproductive performance, physiological response, health and immunity, milk quality and quantity, cashmere production, meat quality and carcass characteristics, and genetic adaptation as affected by heat stress.

### 2.3. Data Collection

**Figure 1:**
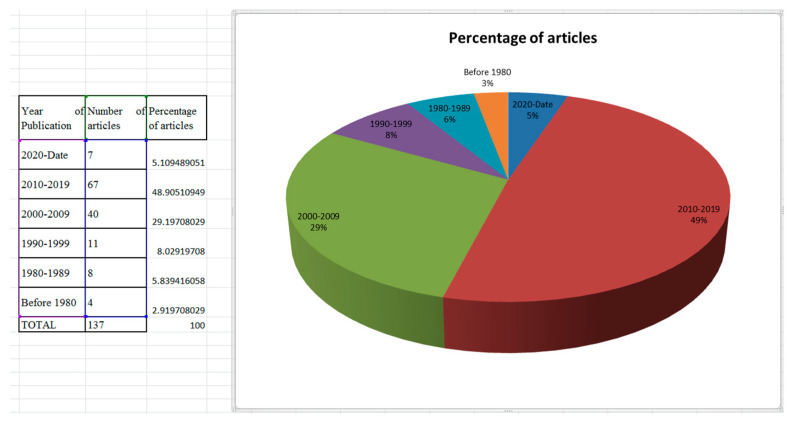
Year of distribution of the articles in this review.

This review was semi-structured because of the integrative and broad nature of the subject matter, which made it difficult to conduct a comprehensive holistic assessment. The information was derived from key academic articles found in the ResearchGate, Google Scholar, Web of Science, and Sci-hub databases (Table 1). Article files were downloaded from these sites following critical studies, analytical brainstorming by all authors, and the production of this review. To ensure optimal coherence in the search and to prevent duplicate articles, we adopted a single database because of the extensive scope of the reviewed literature. Keyword queries were developed for each of the 9 parameters as follows: behavioral response, growth and development, reproductive performance, physiological response, health and immunity, milk quality and quantity, meat quality and carcass characteristics, cashmere production, and genetic adaptability. These questions were created through expert consultations to ensure a comprehensive coverage of the literature. To address the current state of goat production in the event of heat stress, the highest percentage of the reference materials used was published between 2010 and 2024, with a few dated before 2010 to enable authors to understand the trend in the subject matter over the past decades. Figure 1 shows the year of distribution of the articles used in this review.

### 2.4. Reproductive Performance

The effects of heat stress on both male and female reproductive performance were also discussed. The aspects of the male reproductive functions considered included sperm quality and quantity, sperm motility, sperm count, and libido. Testicular degeneration and spermatogenesis were also observed. Similarly, the information on female reproductive characteristics was sourced from various published articles, including estradiol levels, follicular estradiol concentration, aromatase activity, gonadotropin levels, luteinizing hormone (LH) receptor levels, fertility, embryo development, ovulation, and conception.

### 2.5. Meat Quality and Carcass Characteristics

Meat quality and carcass characteristics are influenced by the feed, breed, and environment. The physical characteristics of meat, including color (lightness, redness, and yellowness), water-holding capacity, and cooking loss, are discussed. Chemical characteristics, such as pH, protein, glycogen, and fat content, are also identified. Sensory parameters, including juiciness, texture, and taste, are discussed in this review as well. The information was gathered from published literature from the various sites previously mentioned.

## 3. Effects of Heat Stress on the Production of Goat

### 3.1. Behavioral Responses of Goats to Heat Stress

Although there have been many published articles on the effects of thermal stress on productive and reproductive indices [8,9,13,18], there is a lack of published information regarding the behavioral changes goats undergo under heat stress. Nevertheless, animals exhibit diverse behaviors in response to heat stress, which can offer valuable insights into the methods and timing of cooling. The spectrum of behavioral reactions impacts heat transfer between an organism and its surroundings through reducing heat acquisition by radiation and enhancing heat dissipation through convection and conduction [20]. Goats exhibit many behavioral reactions during heat stress, such as assembling in shaded areas [11], excessive salivation, rapid breathing with an open mouth, reduced food consumption, and increased water intake [21]. In extreme instances of thermal stress in goats, a lack of coordination, palpitating, and lying down of animals may be observed. Furthermore, desert-dwelling animals typically employ nocturnal behaviors (being very active during the night) to mitigate the impact of high temperatures. The act of seeking shelter in the shade is a blatant behavioral adjustment. In the absence of shade, animals adopt a vertical posture relative to the sun to minimize the surface area available for thermal exchange [22]. Animals can adjust their posture by standing or spreading out to increase the surface area for heat loss, and they may decrease activity as well [23]. During extreme heat stress, animals dampen their body surfaces with water, saliva, or nasal secretions [11]. Animals can respond to stimuli through physiological or behavioral responses; nevertheless, they commonly exhibit a combination of both [24]. For example, goats exhibit reduced urine and fecal production when subjected to heat stress [25,26]. The decreased frequency of urination may be attributed to heightened respiratory and cutaneous cooling processes, potentially resulting in significant dehydration and a subsequent reduction in the frequency of urination [26]. Furthermore, a decrease in the frequency of defecation may serve as an adaptation mechanism for these animals to retain body water [26].

During high temperatures, grazing ruminants lower their grazing activity, prefer to rest to minimize movement, and seek shelter in shaded areas [11,18]. Standing and resting are behavioral adaptations that help prevent excess heat from the ground and promote efficient heat dissipation. A study by Shilja et al. [26] and Carolyn, [24] found that goats exposed to heat for 4–8 h a day over 18 days exhibited an increase in standing time (445 vs. 390 min) and a decrease in lying time (50 vs. 90 min) in contrast with the control goats. The interaction between strain and nutrition leads to nutritional insufficiency, owing to a significant decrease in feed intake caused by heat stress [27]. Heat stress directly affects the eating center of the hypothalamus, resulting in a hormonal response that may reduce metabolic rate [23,27]. Animals experiencing heat stress reduce their food consumption to minimize the generation of metabolic heat, as the heat generated during digestion is a significant contributor to heat production [28]. Furthermore, heat stress [29] leads to a 30% increase in maintenance requirements. Additionally, current energy intake is insufficient to meet daily requirements, resulting in noticeable body weight loss [30]. Heat-induced animals experience a comparable bioenergetic state, although not to the same degree as the negative energy balance seen in early lactation. The negative energy balance leads to a range of metabolic and hormonal alterations. Most of the detrimental effects of heat stress on production, animal health, and reproductive indicators are likely caused by a decrease in the energy balance [31].

As reported in the literature, animals exposed to heat stress showed a significant reduction in both feed intake and body weight. Goats have been found to experience a decline in dry matter intake as a result of thermal stress, as described by Hamzaoui et al. [30] and Salama et al. [32]. Furthermore, goats experience reduced body weight, daily dry matter intake, and weight gain when subjected to heat stress [8]. The decrease in body weight under heat stress could be related to the increase in the energy used for heat discharge by respiratory evaporation, which consequently leads to a decrease in the quantity of water usable for storage [8,33]. Heat stress causes a 30% reduction in dry matter intake of dairy goats [34]. Researchers recommend using a variety of nutritional modifications to the diet of producers/farmers to attenuate the impact of heat stress. These changes include maintaining feed intake, increasing nutrient density, and minimizing the adverse effects of heat stress [27]. Water is a vital nutrient that is necessary for sustaining life and plays a crucial role in various physiological functions that are critical for the optimal functioning of small ruminants. Water is crucial for regulating body temperature, promoting development, facilitating reproduction and breastfeeding, aiding digestion, facilitating nutrient exchange and transport in circulation, eliminating waste materials, and maintaining the thermal balance [2]. Water requirements are regulated by factors including dry matter intake, environmental temperature, and various sources of water loss such as body evaporation, urine, feces, and milk. Unlike feed ingredients, water is frequently disregarded when it comes to ensuring the optimum output of ruminant animals, especially those reared in hot conditions. Small ruminants are susceptible to significant water scarcity under various environmental conditions such as drought, transportation issues, and grazing in remote areas without access to water sources. In dry regions, water needs are particularly high owing to intense heat and solar radiation [2]. Goats exhibit exceptional resistance to drought conditions. Goats are generally more efficient at conserving water than sheep, likely because of their browsing diet [18]. Some goat breeds, such as Black Bedouin and Barmer goats, can survive with watering every four days [12]. Desert goats in indigenous systems may only be sprinkled every three to six days under water scarcity [35]. Goats subjected to heat stress exhibit an exponential rise in water intake [34,36]. Heat-stressed goats mostly utilize increased water intake to enhance heat dissipation through sweating and panting. Heat-stressed goats experienced three times more water evaporation from water input than control goats [30].

### 3.2. Effects on Growth and Development

According to Geraseev et al. [37], body growth refers to the enlargement and functional maturation of tissues and organs from conception to maturity. This process involves synthesis and cellular metabolism and is influenced by genetic factors, hormones, metabolism, and the environment. Grant [38] noted that prenatal growth occurs rapidly and exhibits exponential growth in every animal species. During the early stages of gestation, fetal growth is limited and determined by genetic factors specific to the species. However, in the final trimester of gestation, fetal growth accelerates significantly and is heavily affected by the mother’s nutritional intake. Da Souza et al. [39] and Araújo et al. [40] stated that maternal factors significantly influence postnatal growth, particularly during the initial sixty days of lactation. The most susceptible phase in an animal’s life occurs before and swiftly following birth, which is referred to as the perinatal period. The perinatal mortality period for small ruminants occurs from two (2) months of gestation to three (3) weeks after birth [41]. Neonatal mortality is influenced by various variables, including nutritional causes, such as inadequate consumption of colostrum, viral and parasitic disorders, environmental conditions, and the invasion of predators. 

Rapid and sufficient growth is an essential economic factor for meat production. Producers strive to enhance this aspect, as animals with greater weight gain require fewer days to attain the optimum slaughter weight. This approach is more cost-effective and economically feasible because it accelerates the production rate and reduces production costs [42]. Several variables such as genetics, environment, sex, weight, birth type, health, and quantity and quality of the food provided influence body growth [40]. Birth season plays an important part in the development of animals [43]. The factors that contribute to the influence of the season of birth on the weight variation of animals include changes in management practices and feeding methods, the use of different breeders, alterations in the genetics of the flock, and changes in climatic conditions such as rainfall, temperature, and humidity. These factors significantly impact the animals and the quality and accessibility of pastures [40]. In a study conducted by Phillips [44], it was discovered that goats born during the rainy season had a greater birth weight and reached the weaning stage at a faster rate in contrast to goats born during the dry season. This phenomenon can be attributed to a compensatory growth phase, during which all animals had access to ample grass and supplementation, resulting in optimal growth conditions. Shaat et al. [45] conducted a study on crossbred goats to examine their reproductive features and growth. The study found that the year of birth had a substantial impact on the weight of goats at birth, as well as at 56, 84, and 112 days of age. This highlights that environmental conditions can directly impact the physiological functions of an animal’s body, and indirectly affect the availability and quality of food or increase the occurrence of diseases. These factors significantly influence animal productivity (Table 2).

### 3.3. Effects of Heat Stress on Reproductive Performance of Goats

Ruminants, similar to all mammals, are homeothermic. They possess the ability to control their body temperature through physiological, behavioral, and metabolic mechanisms such as thermolysis or thermogenesis, especially under extreme ambient temperature conditions [50]. These mechanisms allow animals to attain thermal comfort when they reach a state of thermal neutrality with minimal energy use to maintain temperature balance, optimizing their ability for production and reproduction [51]. However, when the thermal comfort zone is not achieved, the interaction between the animal and its surroundings leads to heat stress, which hampers the productive and reproductive development of the animal, leading to economic losses [52]. Heat stress triggers the thermoregulatory region in the central nervous system, which subsequently stimulates compensatory mechanisms like thermolysis (peripheral vasodilation, increased respiratory rate, decreased food intake, and increased water intake). These mechanisms help animals dissipate heat via conduction, convection, and radiation. The efficacy of heat dissipation depends on the sweat glands’ activity in an explicit mechanism. When males are exposed to thermal stress, there is a rapid decrease in the volume of ejaculated fluid, sperm motility, sperm count with anomalies, and libido [53]. Hafez [54] observed that when the environmental temperature is high, it causes an elevation in body temperature, which, in turn, can result in testicular degeneration and a decrease in the proportion of healthy and viable sperm in the ejaculate. Silva [51] also reported a clear correlation between environmental temperature and semen pH. When animals are exposed to an exponential temperature of 33 °C and remain in this stressful condition for an extended period, they may experience fatal testicular degeneration. Little research has been undertaken on the effects of the environment on particular functional anomalies of the reproductive organs in female goats [55]. However, the underlying mechanisms are believed to be similar to those observed in cows. 

Da Silva et al. [56] examined the impact of different reproductive conditions (parous and nulliparous) in climate-divergent environments (dry or wet season). They found that the quantity and quality of embryos collected from super-ovulated Boer goats were greater in the rainy season compared to the hot season when the donors were nulliparous. However, this pattern was not observed in parous donors. This can be attributed to the adaptability of the climate, which does not impose any limitations on reproductive capacity. Goats that have acclimated to dry and semi-arid regions have a bifurcated scrotum as an identifiable adaptation to the climate in these areas [57]. Additionally, they exhibit variations in spermatogenesis when exposed to suboptimal temperatures. In their study on goats, Silva et al. [58] found a decrease in sperm concentration during the hottest season of the year. According to Salles [59], during the dry season, when the temperature is elevated, there is a decline in sperm quality, which is characterized by alterations in the proportion of motile spermatozoa and an increase in sperm maladies. Testosterone levels increased during this period. Females lack these anatomical configurations and exhibit the physiological impairment of reproductive function upon exposure to high-temperature swings. Heat stress has a direct impact on the reproductive capacity of animals by suppressing the release of gonadotropin-releasing hormone (GnRH) in the hypothalamus, which is a part of the hypothalamic–pituitary–gonadal axis. The anterior hypophysis inhibits the production of follicle-stimulating hormone (FSH) and luteinizing hormone (LH), whereas in the gonads, it alters the stimulating action of gonadotropin secretion of sex steroids [60]. Hence, reproductive efficiency is impacted [61], as spermatogenesis is regulated by the neuroendocrine system and influenced by the scrotal testicular thermoregulation mechanism. Activation of the hypothalamic–pituitary–adrenal (HHA) system in females leads to a decrease in the excretion of gonadotropins (LH and FSH) and a reduction in estrogen production. This, in turn, can cause reproductive disorders such as the inability to detect estrus (silent estrus) and problems with oocyte development, fertilization, and insemination. Shehab et al. [62] observed that heat stress caused a decrease in the diameter of the dominant follicle. This size reduction negatively affects oocytes’ developmental capacity and granulosa cell quality. As a result, the reproductive outcomes are negatively impacted, especially in high-producing dairy cows exposed to heat stress conditions. Embryo mortality occurs because of the secretion of CRH, which inhibits the release of LH by acting on GnRH. This leads to failure in oocyte release and alterations in the endometrial cells, ultimately resulting in pregnancy loss. According to Corassin [63], cows exposed to rain for a specific period had a five-fold higher likelihood of conceiving than cows exposed to rain during summer. 

**Figure 2:**
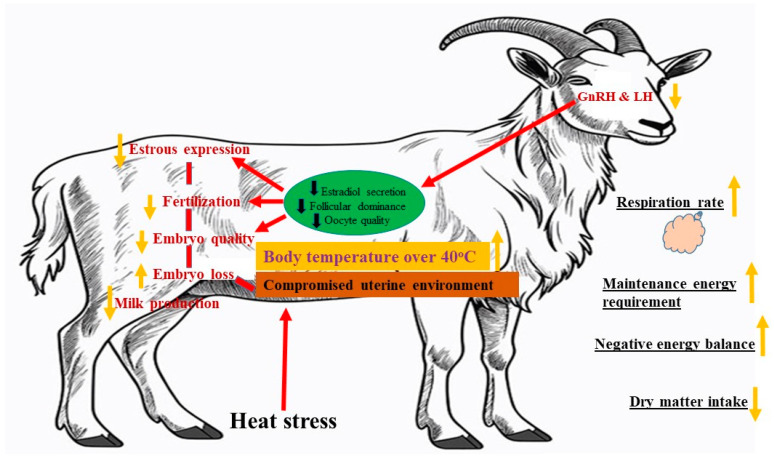
Effects of heat stress on female reproductive performance. Modified from [71].

Goats grown in tropical climates are continuous breeders. However, when there is limited food available, they often experience long periods of inability to reproduce and have fewer chances of ovulation. This leads to decreased fertility and lower rates of giving birth to multiple offspring [64] (Figure 2). Several researchers [65,66,67] discovered that Ossimi and Rahmani sub-tropical sheep breeds, as well as their crosses (such as Suffolk × Ossimi crossbred and various crosses between Finn), exhibited reduced fertility in May (spring) compared to mating in September (autumn) and January (winter) in Egyptian conditions. This decrease in reproductive performance has been attributed to functional issues experienced by both males and females copulating during periods of heat stress [68]. The results obtained by [64] regarding seasonal effects on the conception rate of goats aligned with those of Marai et al. [69], who reported that the summer mating season, in general, showed the lowest pregnancy rate. In addition, these researchers [66,67], observed an inverse relationship among conception rate, ambient temperature, and daylight length, in Ossimi, Rahmani, and Ossimi × Suffolk ewes, respectively. Heat stress in goats leads to a decrease in estradiol levels, follicular estradiol concentration, aromatase activity, gonadotropin, and LH receptor levels, which, in turn, causes delayed ovulation [54,70]. Additionally, temperatures exceeding 32 °C might lead to thermal stress, particularly when coupled with high humidity levels.

### 3.4. Heat Stress Effects on Physiological Responses

Elevated ambient temperatures can have numerous physiological repercussions, leading to significant economic losses in the goat business. These variables include impairments in the reproductive system, increased oxidative stress, impaired enzyme activity, electrolyte imbalances, disruption of endocrine balance, declined feed intake, and poor meat quality [72,73,74]. Physiological measures, such as respiration rate, heart rate, and rectal temperature, exhibit a prompt reaction to heat stress [72], thus reflecting the degree of animal discomfort or comfort. The respiration rate, heart and/or pulse rate, and rectal temperature are often used as indicators of physiological adaptation to heat stress in small ruminants [1,73,75]. The primary indicators of thermal stress in goats are elevated body temperature and respiration rate, as highlighted by [25]. It is worth noting that the intricacy and range of physiological changes mediated by thermal stress may differ depending on the animal’s species, the individual animal, and their hormonal state.

### 3.5. Effects of Heat Stress on Health and Immunity

Despite impacting the production quality, heat stress also affects the immunological responses of goats. Sophia et al. [76] observed that heat stress reduced goats’ innate immune response which is typically regarded as the initial defense mechanism. Nevertheless, several studies have highlighted the ineffectiveness of goats’ inherent immune responses in the absence of heat stress [77]. A decrease in immunoglobulin secretion results in a compromised adaptive immune system, which increases the likelihood of parasite infestation [78]. Hirakawa et al. [79] observed that severe temperatures led to reduced lymphocyte production and inhibited the phagocytic functions of leukocytes in goats. Furthermore, Yadav et al. [80] found that heat stress had a negative impact on antibody synthesis in goats, explicitly affecting the production of IgM and IgG. TLR2, TLR8, IL10, IL18, TNFα, and IFNβ have been recognized as significant inflammatory indicators for measuring the influence of heat stress on the immune system in goats [81]. Heat stress significantly impacts an animal’s immunological response, making it vulnerable to deadly diseases. A study on Malabari goats found that interleukin 18 (IL-18), tumor necrosis factor-α (TNF-α), interferon-β (IFN-β), and IFN-γ are reliable immunological markers for evaluating changes in the immune response caused by heat stress [82]. The mRNA expression levels of all three genes were markedly reduced under heat stress in goats. Similarly, Madhusoodan et al. [83] observed a substantial decrease in the expression of hepatic IL-2, IL-6, IL-18, TNF-α, and IFN-β mRNA in heat-stressed Salem Black goats. They ascertained that these genes may serve as indicators of heat stress in this strain. Collectively, it is evident that goats reduce their productivity to adapt to unfavorable conditions, thereby jeopardizing the economic stability of destitute and marginalized farmers whose only source of revenue depends on goat farming. Hence, it is imperative to prioritize the implementation of welfare measures to maintain goat production under heat stress.

### 3.6. Effects of Heat Stress on Milk Production and Quality

The production and quality of goat milk are influenced by various factors such as the type and quality of the diet, breed of the goats, duration and timing of lactation, climatic variations, and the combined effects of these parameters in different environmental scenarios of each country or region [84]. Goats are usually considered robust animals. However, they undergo physiological changes when exposed to hot regions with elevated temperatures, humidity, and radiation. These changes include increased skin temperature, heightened rectal temperature, higher respiratory rate, reduced feed intake, and decreased production levels [85]. When animals experience temperature stress, they exhibit decreased food and water consumption and reduced milk production [86]. Caloric stress results in reduced meat and milk production as well as reproductive and dietary problems [87]. These processes occur due to the influence of air temperature, relative humidity, sun radiation, wind, and the strength and duration of the stressor agent [85]. In addition, Saanen goats experience a decline in feed intake and an increase in daily water consumption when exposed to the temperature of 32.5 °C in a bioclimatic chamber [88]. However, their milk output remained similar to that of the group maintained in thermal comfort. In research conducted by Juarez [89] on Saanen, Anglo-Nubian, and Alpine goat breeds in tropical climates, it was found that milk production was low, and certain milk components, such as fat and total solids, were reduced compared to when these breeds were raised in temperate climates. This can be attributed to inadequate diet and elevated air temperatures. Goat dairy herd productivity may be reduced as a result of adverse climatic conditions in semi-arid regions [90]. In their research, Darcan and Güney [91] demonstrated that milk production increased by 21% in a group of goats subjected to spraying and ventilation compared to the control group that received neither spraying nor ventilation. The climate has a significant impact on both animal welfare and productivity. It is a limiting element in the economic exploitation of animals for various reasons [39]. A different study involving Saanen, Anglo-Nubian, and Alpine goat breeds revealed a decrease in milk production and a reduction in the concentration of certain components, such as fat and total solids, in the milk of goats raised in tropical climates compared with those raised in temperate climates. This can be attributed to elevated air temperature and insufficient diet in tropical climates [89]. In situations with elevated temperatures, when the production of heat exceeds its dissipation, all heat-generating sources, particularly food consumption and metabolism, are suppressed [92].

### 3.7. Effects of Heat Stress on Meat Quality and Carcass Characteristics 

Heat stress influences meat quality, carcass physical traits, and organoleptic qualities of sheep and goats. The diminution of meat quality attributes, such as pH, color, texture, and moisture, is commonly known as dark cutting or dark-firm drying, characterized by high pH and low glycogen levels in the flesh [93]. Rana et al. [94] elucidated the potential negative impact of diverse physiological phenomena on meat quality. First, heat stress triggers a sufficient adrenaline surge that facilitates the dilation of blood vessels in the periphery and the hydrolysis of muscle glycogen. Prolonged exposure before slaughter may result in elevated pH levels and darker meat. Additionally, when an animal engages in physical activity and experiences hyperthermia before being slaughtered, the elevated temperature and anaerobic metabolism result in earlier and more intense rigor mortis. Furthermore, elevated temperatures induce dehydration in animals without sufficient water, thereby affecting meat quality. This is manifested by darker hues owing to the contraction of myofibrils, as well as reduced weight loss during the cooking process owing to its dryness. Sheep and goats slaughtered at an ambient temperature of approximately 35 °C exhibited certain differences in meat quality compared with those slaughtered at 21 °C. The former group exhibited a higher pH level of 5.78 compared to 5.65 in the latter group. Additionally, the former group had a myofibrillar fragmentation index of 86.88%, whereas the latter group had a value of 85.59%. Additionally, their meat had lower color values (lightness, redness, and yellowness) and contained less juice (35.74 g/cm^2^ vs. 36.84 g/cm^2^). These findings suggest that variations in meat quality are primarily influenced by seasonal temperature [74]. Heat stress (HS) at a temperature of 27 °C and humidity of 88% significantly impact various factors related to pre-slaughter and carcass of goats. This includes an increase in cooking loss (43% compared to 36%) and pH levels (6.30 compared to 6.16). Additionally, the by-products of heat-stressed goats show an increase in the weight of the blood (173 compared to 437 g), pluck (370 compared to 573 g), heart (32 compared to 50 g), spleen (20 compared to 43 g), and kidney (20 compared to 43 g) compared to non-heat-stressed goats [95]. 

Meats that have a high ultimate pH and cause harm to the intestinal tissue of live animals have become widely accepted as a safety concern for both meat and its derivatives. Psychrophile bacteria, capable of growing at refrigerator temperatures and causing product deterioration, are hindered by the optimum pH (5.6) of meat. However, they can thrive in meat with a high binding pH, including *Acinetobacter* and *Altermonas putrefaciens* [96]. Conversely, HS causes tissue injury and rubor in the guts of ruminants, leading to increased permeability of the intestines and attachment of bacteria to the intestinal lumen [97]. Elevated levels of stress hormones such as catecholamines and glucocorticoids can alter the function of the intestinal barrier and microbial ecology in ruminants [98]. Heat stress negatively affects the structural integrity of the intestines and causes an increase in their permeability to endotoxins and the movement of pathogenic bacteria (such as *Salmonella*, *Escherichia coli*, and *Campylobacter*) into the bloodstream [99]. The presence of intestinal bacteria in the muscle and liver, dependent on animal health, can result in foodborne diseases in humans. Therefore, HS threatens meat safety, particularly in low- to medium-input production systems in developing countries, where ruminant production plays an integral role in maintaining food security.

### 3.8. Effects of Heat Stress on Cashmere/Wool Production and Quality

The economic features of cashmere, such as yield and length, are influenced by factors such as variety, age, nutritional level, and environment [100]. In 1987, Australian researchers discovered that wild goats undergo seasonal variations in wool according to environmental conditions [101]. Variations in the duration of light exposure affect the growth rate, length, and diameter of cashmere in goats. Research has demonstrated that light’s impact on cashmere growth corresponds to the animals’ biological cycles. Variations in photoperiods affect animal hormone release and further influence the cyclical growth and maturation of hair follicles [102]. The release of melatonin is a major contributing factor. The pineal gland limits the release of melatonin during the day, produces significant amounts of melatonin during the night, and directly impacts the growth of cashmere [103]. Experiments have demonstrated that exposure to shorter periods of light results in elevated levels of melatonin production and stimulates the growth of cashmere. Studies by these researchers [104,105] showed a considerable augmentation in the length of cashmere fibers and the production of cashmere in the light control group, consistent with prior research on goats.

### 3.9. Genetic Adaptations of Goats under Heat Stress

Heat stress significantly impairs livestock output, although its effects differ among animals depending on the species and strain. The genetic capacity of an animal is the determining factor for its ability to resist adverse environmental conditions [13]. Aleena et al. [106] and Madhusoodan et al. [83] have documented the existence of differences among goats in their response to thermal stress and tolerance efficiency. These variations are based on molecular responses, that is, alterations in heat shock protein 70 (HSP70), TLR2, and TLR8. This research allows for the detection and measurement of biomarkers related to heat stress, which could help achieve long-term genetic objectives for developing breeds appropriate to agroecological zones. Animals demonstrate several adaptive mechanisms when subjected to elevated temperatures [107]. This area is a developing branch of study focused on investigating and confirming the potential indicators of heat stress. Furthermore, it is imperative to prioritize the identification of welfare parameters. The body condition score, hair coat condition, resting position, panting score, and behavioral responses are recognized welfare markers in goats [108]. However, it is imperative to establish solid empirical foundations before integrating indicators into a welfare evaluation process that is valid, reliable, and attainable. Integrating molecular genetics into animal breeding programs has led to significant development and improvement in animal breeding practices. Nevertheless, most of these studies concentrated on features related to productivity. Given the increasing apprehension regarding climate change and its effects on livestock productivity, breeders and policymakers have begun to advocate developing heat-resistant breeds.

Indigenous animals are undeniably more suited to withstand climatic challenges than exotic or crossbred animals, which results in improved well-being metrics. Indigenous breeds exhibit varying degrees of resilience to climate change [96]. Aleena et al. [106] examined the resistance ability of three local goat breeds, Osmanabadi, Malabari, and Salem Black, when exposed to heat stress. After conducting a thorough analysis of several physiological, behavioral, and molecular evaluations, it was proven that Salem Black goats exhibited enhanced adaptation to heat stress compared to Osmanabadi and Malabari goats. The variations in the heat resistance capabilities of indigenous goat breeds highlight the necessity of evaluating the thermotolerance capacity of all native goat breeds and identifying superior breeds with enhanced ability to tolerate extreme heat. The Salem Black indigenous goat breed demonstrated superior adaptation to heat stress compared to the other two indigenous goat breeds native to distinct agroecological zones [96]. The study’s remarkable discovery was that Salem Black goats exhibited strong performance, even in a test environment that differed from their habitat. Therefore, it is vital to prioritize the development of agroecological zone-specific breeds to ensure sustainability despite climate change conditions. The genetic underpinnings of thermotolerance entail intricate mechanisms resulting from a combination of several genes and phenotypes [109]. Nevertheless, this element has received limited attention and is currently the subject of investigation by scientists worldwide.

Yakubu et al. [110] examined the single nucleotide variants in the MHC class II DRB gene and analyzed how they are linked to thermo-physiological features in three prominent goat breeds in Nigeria, namely the West African Dwarf (WAD), Red Sokoto (RS), and Sahel (SH) goats. They identified 14 alleles, of which 7 alleles were significantly associated with heat tolerance. In addition, they also observed that the SH and RS goats exhibited superior thermal adaptation to the hot and humid tropical climate in Nigeria compared to WAD goats [110]. Khan and Dige [111] investigated the importance of superoxide dismutases-3 (SOD-3) in the context of thermal stress. They examined the genetic variations in SOD-3 and analyzed how they relate to heat tolerance features in three goat breeds (Black Bengal, Ganjam, Raighar). The researchers uncovered three single nucleotide polymorphisms (SNPs) in the SOD3 gene that had a substantial effect on important physiological factors and specific plasma biochemical variables such as total proteins, albumin, bilirubin, creatinine, ALT, ALP, glucose, and triglycerides [111]. Thus, the correlation between genetic diversity in the SOD-3 sequence and the characteristics related to the response to heat stress could possibly be regarded as a key DNA marker for the selection of traits related to tolerance to high temperatures. In addition to SNP markers, certain genes may serve as genetic markers for goats’ tolerance to heat stress due to their mRNA expression profiles. A study conducted by Shilja et al. [26] evaluated the ability of Osmanabadi goats to adjust to heat stress and nutritional stress throughout the summer season. Researchers have found that the expression of the adrenal HSP70 gene could serve as a promising biomarker in this context. The study discovered that the mRNA levels of HSP70 were elevated in the adrenal glands of goats subjected to both heat stress and nutritional stress compared to goats exposed mainly to heat stress or nutritional stress. According to researchers, the elevated production of HSP70 is a result of the hyperactive adrenal cortex, which is responsible for producing cortisol, a crucial indicator of stress. In addition, Angel et al. [112] identified growth hormone (GH), growth hormone receptor (GHR), insulin-like growth factor-1 (IGF-1), leptin (LEP), and leptin receptor (LEPR) as optimal biomarkers for evaluating the growth potential of Malabari goats under heat stress conditions. The mRNA expression of all the genes described above was dramatically decreased in goats exposed to heat stress.

**Figure 3:**
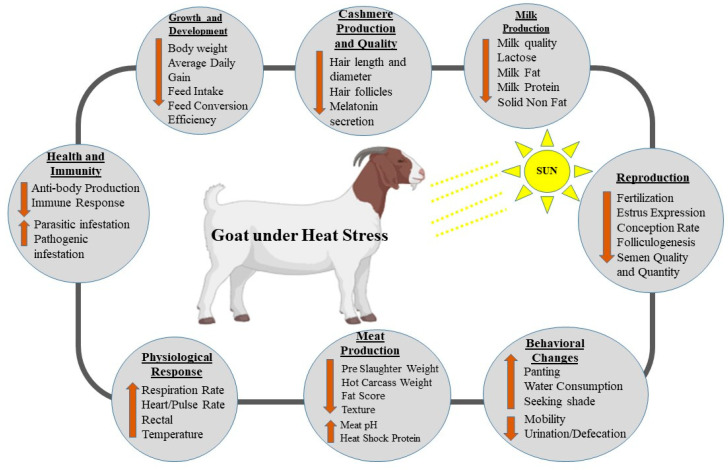
Summary of the effects of heat stress on goat production. Created with the web-based BioRender tool (BioRender.com).

In summary, heat stress affects both the productive and reproductive capacity of goats. The reproductive capacities of goats are adversely affected by a lower estrus cycle, lower folliculogenesis, lower conception rates, and lower fertilization rates resulting from reduced semen quality and quantity from male goats. Physiological, behavioral, growth, and developmental parameters were also affected. Health and immune functions are compromised during heat stress, making the animals susceptible to diseases. Lastly, productive characteristics, such as milk quality and quantity, meat quality, and cashmere, are drastically reduced when goats are exposed to heat stress. This results in low productivity and socioeconomic loss. Figure 3 illustrates the summary of the effects of heat stress on goat production.

## 4. Mitigation Strategies in Alleviating Heat Stress

**Figure 4:**
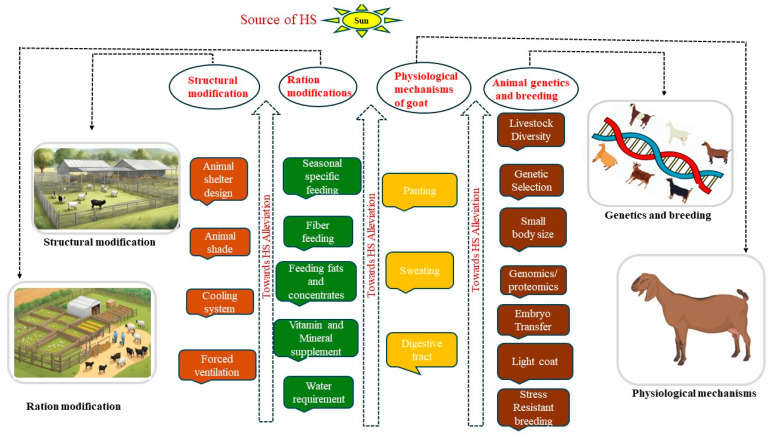
Mitigating approaches towards heat stress. Created with the web-based BioRender tool (BioRender.com).

From the previous sections, it can be deduced that exposure to heat stress (HS) generates diverse modifications in the biological functioning of goats. These changes include decreased feed intake, disturbance in the regulation of water, protein, energy, and mineral levels, and alterations in enzymatic reactions, hormonal secretions, and blood metabolites. Therefore, small ruminant producers/owners should adopt an array of approaches to mitigate the detrimental effects of heat stress (HS). These methods include establishing shelters, using strategic feeding and grazing techniques, ensuring water availability, managing handling time, utilizing fans and evaporative cooling systems, and carefully choosing the location for housing the animals [15,36,113]. Moreso, the inherent physiological traits of goats coupled with modern genetic breeding systems enable animal and goat farmers to alleviate heat stress in goat production. Figure 4 shows the mitigation strategies for counteracting heat stress.

An essential adaptation component is an organism’s inherent genetic capacity to endure and thrive under challenging circumstances [114]. Climate-adapted animals have developed distinct physiological and behavioral mechanisms to regulate body temperature and maintain homeostasis, as illustrated by [115,116]. For instance, sweating enables animals to expel heat from their bodies via evaporation, while heaving stimulates airflow throughout the respiratory system, facilitating heat dissipation. Animals also employ the strategy of decreasing their metabolic activity and heat generation to adapt to HS [117]. Goats possess physiological traits that make them well-suited for living in deserts, giving them an edge over some other ruminant species in challenging environmental conditions. Their diminutive physique, short and pointed erect ears, fleece structure, light coat, and exceptional digestive efficiency allow them to withstand harsh weather conditions [118].

Shading is the easiest way to mitigate the effects of intense solar radiation, and it may be applied in a wide range of situations. A small proportion of sheep and goat folds may be housed at all times, whereas the majority are kept indoors at night and during times when grazing is impossible [119]. In semi-intensive systems, it is not feasible to employ shades, fans, or evaporative cooling methods since sheep and goats are allowed to graze in open areas for the majority of the day. Therefore, other solutions, such as portable shades, must be implemented to extenuate the negative impacts of heat stress [3]. Providing animals with access to shade throughout the summer is a basic, relatively inexpensive, and effective method for reducing heat stress [18,120]. Allowing sheep and goats to seek shade results in enhanced weight gain, milk production, and reproductive performance [121]. Additionally, it decreases goats’ rectal temperature and respiration rate [122]. According to Muller et al. [123], a suitably built shade structure can decrease the heat load by 30–50%. The shelters are simple and basic. Trees and shrubs can protect animals against solar radiation [124] and are often the most economical option. In the absence of natural protection, sheep and goat producers usually use Quonset huts, plastic calf hutches, poly domes, or carports as alternative shelters for grazing animals. Additionally, hay or straw blinds, sheet metal painted white on top [125], and aluminum sheets [126] are the most efficient and cost-effective materials.

Modifying rations can effectively mitigate the adverse effects of HS. These modifications encompass alterations in feeding schedules (e.g., feeding during cooler hours and adjusting feeding intervals), grazing time, and ration composition. Adjustments in ration composition may involve modifying dietary fiber content, incorporating high-quality fiber forage, increasing energy density through the addition of protected fat supplements, and utilizing feed additives, such as buffers (sodium bicarbonate), niacin, antioxidants, and fungal culture (yeast culture). In summer, animals typically exhibit altered feeding patterns and consume larger quantities of food during cooler parts of the day [27]. Hence, providing animals with feed during cooler periods of the day helps them maintain their usual food consumption and avoids the simultaneous emergence of high metabolic and climatic heat stress [127]. Additionally, providing animals with feed at shorter intervals aids in reducing daily variation in ruminal metabolites and optimizes the efficiency of feed utilization in the rumen [128].

Another factor to consider when reducing HS is the duration of grazing time. During periods of high temperatures, animals reduce their grazing duration and spend more time seeking shelter in shaded areas, particularly during peak hours of the day. They engage in grazing activities during the temperate periods of the day, specifically before sunrise, before dawn, and at night [4]. Proper diet modification throughout the high-stress period is crucial for attaining optimal animal performance. Reducing the proportion of forage in relation to concentration can lead to rations that are easier to digest and that can be ingested in larger quantities [73]. Providing feed with low fiber content during hot weather is appropriate because the creation of heat is closely linked to the breakdown of acetate, as opposed to propionate, in the body’s metabolism [129]. In general, diets higher in nutrients are preferred during HS [27]. Dairy goats that received a 4% fat supplement throughout the summer exhibited a decrease in rectal temperature. The addition of soybean oil to the diet of goats housed under heat-stress conditions increases the fat content of their milk [34]. Feed additives are being advocated to counter the effects of HS. Antioxidants, such as vitamins C and E, safeguard the body’s defensive approach against excessive generation of free radicals (which are scavenged by antioxidants) during heat stress, thereby stabilizing the animal’s health condition [3]. Ayo et al. [130] and Ghanem et al. [131] discovered that providing vitamin C supplements to sheep and goats effectively reduces the symptoms of HS. The administration of Vitamin E and C supplements also reduces the rectal temperature and respiration rate, as demonstrated by [132]. Additionally, these supplements alleviate heat stress in goats, as observed by [133]. An effective method for minimizing heat stress is to ensure sufficient and cooled drinking water [11,129]. Sheep and goats require more water when exposed to high temperatures. Therefore, it is important to guarantee that animals have constant access to sufficient, clean, cool, and freshwater. This can be achieved by ensuring the presence of sufficient watering devices with appropriate pressure to refill waterers and by increasing the number of water sources in the pasture [129].

Genetic selection for heat tolerance can offer a sustainable means for augmenting feeding and housing adjustments. Functional genomics can be used to identify the selection signatures for thermotolerance. Additionally, productive breeds can be enhanced by crossbreeding with resilient genotypes and incorporating stress-tolerant genes. Moreover, indigenous breeds, especially those originating from the Near East and Africa, exhibit superior acclimatization to elevated temperatures and challenging situations and generally possess a higher capacity to adjust to adverse conditions than foreign breeds [114]. Hence, employing selective breeding to enhance thermotolerance and coupling well-suited ruminant breeds (Table 3) with appropriate locations and production systems is an effective approach for mitigating heat stress in goat farming.

## 5. Conclusions

Goats can thrive in several ecological zones, including temperate climates, owing to their adaptive traits and mechanisms that help them combat heat stress. Goat farming is a key component of global food security. In the current era of persistent global warming, which has led to increased ambient temperature, heat stress poses an urgent threat to food security. Although goats possess many adaptive features, they are not exempt from this challenge in the livestock industry. This review concludes that heat stress induces physiological alterations in animals, leading to increased vulnerability to diseases owing to compromised immunity. Additionally, it causes a substantial reduction in reproductive and productive capacities, resulting in economic losses for livestock owners and other stakeholders across the food chain. Elevated temperatures harm key aspects of goat biology and productivity, including reproduction, immune system function, growth, milk yield, meat quality, cashmere production, and behavioral and physiological responses. These factors are important to the livestock industry. Understanding the repercussions of heat stress necessitates a collaborative and constructive approach to eliminate this threat. Effective ways to mitigate heat stress in goat production include enhancing the physiological resilience of goats, modifying their housing to optimize temperature regulation, providing shade, adjusting their diet and nutrition, and employing genetic engineering techniques to develop heat-tolerant breeds.

## 6. Future Perspective

Continued research is expected to investigate goat behavior in their natural habitat by employing sophisticated tools to obtain extensive knowledge of the subtle aspects of their well-being. Conducting a comprehensive analysis of native breeds and choosing breeds that are uniquely adapted to agroecological zones helps destitute and marginalized farmers select the most suitable breed. Technological advances are essential for developing credible biomarkers to gauge the well-being of goats. Employing genetic technology may result in the development of heat-resistant breeds, guaranteeing sustained agricultural production and revenue for farmers in climate change scenarios. This could facilitate the development of appropriate breeding strategies and enhance goat production efficiency.

## Figures and Tables

**Table 1:** Number and sources of articles cited.

Aspect of Goats Production	Articles Identified	Source
Behavioral response	25	Google Scholar, Sci-hub
Growth and development	12	ResearchGate, Google Scholar
Reproductive performance	21	ResearchGate, Sci-hub, Web of Science
Physiological Response	6	Google Scholar, ResearchGate,
Health and immunity	8	Research Gate, Sci-hub, Google Scholar
Milk quantity and quality	10	Web of Science, Google Scholar, Sci-hub
Meat quality and carcass characteristics	8	Google, scholar, Sci-hub
Cashmere production	6	ResearchGate, Google Scholar
Genetic adaptability	12	Research Gate, Sci-hub, Google Scholar

**Table 2:** Effects of heat stress on growth in animals.

Breed	HS Condition	Body Weight Change	Observation	References
Osmanabadi goats Malabari goats Salem Black Goats	Summer exposure: 73.5 to 86.5 THI Shed feeding: 69.9 to 74.9 THI 45-d feeding	ADG: Osmanabadi: Exposure—39.63 g Shed 48.02 g Malabari: Exposure—25.00 g Shed: 39.29 g Shalem Black: Exposure—21.03 g Shed 34.53 g	Heat stress significantly reduced the body weight gain among all heat-exposed groups, but the reduction in feed intake of the heat stress group was not significant (except for Malabari goats)	[46]
Poll Dorset × (Border Leicester × Merino) lambs Dorper lambs	HS: 28 °C to 38 °C, 40% to 60% RH cyclic TN: 18 °C to 21 °C, 45% to 55% RH 2-wk study	ADG: Dorper: HS—50.6 g TN: 5.95 g 2nd cross: HS—92.3 g TN 101.0 g	Two weeks of cyclic HS had a significant negative influence on feed intake and body weight gain of wool breed lambs (2nd cross), but the impact of HS was not significant for the hair breed (Dorper lambs).	[47]
White Suffolk × Merino × Border Leicester lambs (42 ± 2.0 kg; 7 mo)	HS: 28 °C to 40 °C, 30% to 40% RH TN: 18 °C to 21 °C, 40% to 50% RH	Feed intake: HS 959 g/d TN 1266 g/d	One week of HS significantly impacted 2nd cross lambs’ feed intake compared with the TN group.	[48]
Dorper × Katahdin male lambs (34.6 ± 1.4 kg; 4.5 mo)	Summer: 28.3 ± 4.0 °C, 77.2 ± 5.4 THI Winter: 19.2 ± 2.6 °C, 64.0 ± 3.0 THI 30-d study	ADG: Dorper × Katahdin: Summer 226 g Winter 302 g	The average body weight gain and feed efficiency of the summer group were significantly lower than those of the winter group.	[49]

**Table 3:** Genes associated with heat tolerance in goats.

Breeds	Genes	Function	Reference
Baraki goat	*FGF2*, *GNAI3*, *PLCB1*	Thermo-tolerance (melanogenesis)	[134]
Mexico goat	*HSP-70*	Thermo-tolerant	[135]
Baraki goat	*MYH*, *TRHDE*, *ALDH1A3*	Energy and digestive metabolism	[134]
Uganda goat	*1L10RB* and *IL23A*	Immune response	[136]
Baraki goat	*GRIA1*, *IL2*, *IL7*, *IL21*, *IL1R1*	Nervous and autoimmune response	[137]

## Data Availability

No data were created during the preparation of this article.
